# Meaning-Making Among Family Members of Hospitalized Traffic Accident Victims: A Grounded Theory Study

**DOI:** 10.7759/cureus.115719

**Published:** 2026-09-03

**Authors:** Erick Alexander de Jesús Chamorro Segovia, Karen Vanessa Herrera, Ana Milena Marquina de Reyes, Jazmin del Rocío Lopez, Ottoniel Sanchez Murgas

**Affiliations:** 1 Center for Research and Health Studies (CIES), National Autonomous University of Nicaragua, Managua, NIC; 2 Quality and Patient Safety, Universidad Evangelica de El Salvador, San Salvador, SLV; 3 Faculty of Health Sciences, Universidad Gerardo Barrios, San Miguel, SLV; 4 Public Health, Universidad Evangelica de El Salvador, San Salvador, SLV

**Keywords:** family caregivers, grounded theory, meaning making, qualitative research, road traffic accident (rta)

## Abstract

Background

Traffic accidents represent an important public health problem in Nicaragua, yet national research has largely emphasized quantitative outcomes and has paid less attention to how families interpret these events during hospitalization. This study aimed to understand how family members of hospitalized traffic accident victims construct meaning around the accident during the patient’s hospitalization.

Methodology

We used a qualitative grounded theory design. Ten adult immediate family members who had actively accompanied the patient and assumed caregiving responsibilities participated in face-to-face, in-depth interviews. Participants were recruited according to predefined eligibility criteria. Data collection and analysis proceeded iteratively until additional interviews no longer substantially modified the emerging categories or their relationships. Transcripts were analyzed in ATLAS.ti 9 using open coding, constant comparison, analytical memo writing, and axial coding.

Results

Participants included eight women and two men aged 21-51 years; nine of the 10 accidents involved motorcycles. Analysis generated an emerging conceptual model comprising five interrelated categories: initial coping with the event; family reorganization and immediate overload; attribution of meaning to the accident; bodily vulnerability and empathy with the victim; and social support and the hospital environment. Participants described an initial emotional and somatic shock that rapidly coexisted with caregiving decisions, administrative demands, and family role reorganization. They interpreted the accident through notions of responsibility, vulnerability, injustice, and road risk, while uncertainty regarding disability or amputation intensified distress. Family, community, religious, and healthcare support shaped how participants navigated the crisis. Across these categories, meaning-making emerged as a process through which relatives progressively incorporated the accident into their understanding of family roles, relationships, and expectations for the future. Although caregiving experiences were predominantly described by women in this sample, its composition does not support broader conclusions about gender distribution.

Conclusions

These findings suggest that the effects of hospitalization after a traffic accident extend beyond the injured person and involve the wider family network. They generate hypotheses regarding the potential relevance of clear communication and family-centered support during acute care, which should be evaluated in future research.

## Introduction

Traffic accidents remain a major public health problem worldwide, causing approximately 1.25 million deaths and between 20 and 50 million nonfatal injuries each year, many of which result in permanent disability [1]. Their burden is particularly pronounced in low-income countries, which account for a disproportionate share of road traffic deaths despite having fewer registered vehicles [2]. In Nicaragua, available epidemiological evidence indicates that traffic accidents frequently involve young and middle-aged men and that motorcycles represent an important mode of transportation among those affected [3]. Technology-based approaches have also been developed to strengthen motorcycle security and real-time vehicle monitoring, including GPS-GSM tracking systems [4]. Although these systems primarily address vehicle tracking and security rather than injury outcomes, they reflect broader attention to motorcycle-related risks and their management. This context further supports examining the consequences of motorcycle crashes for injured persons and their families.

Beyond mortality and physical injury, traffic accidents can disrupt family relationships, routines, responsibilities, and expectations about the future. Research on traffic accidents in Nicaragua has predominantly followed a quantitative orientation and has devoted limited attention to relatives’ experiences during hospitalization. Yet, an accident requiring inpatient care rarely affects only the injured person. Family members may confront acute emotional distress, caregiving demands, financial disruption, changes in daily routines, and uncertainty regarding recovery. International evidence has documented secondary traumatic stress, dissociative symptoms, somatic manifestations, and broader family consequences following exposure to traumatic events [5,6]. Trauma may also become central to relatives’ personal narratives, influencing how they understand relationships, identity, and future possibilities [7,8].

Meaning-making provides a useful conceptual perspective for examining these experiences. Traumatic events can challenge previously held assumptions, expectations, and interpretations of everyday life, creating a need to understand what occurred and to incorporate the event into an altered sense of reality [9]. This process does not occur exclusively at the individual level. Within families, meaning may be negotiated through shared narratives, changing relationships, caregiving responsibilities, and collective responses to uncertainty [7,8]. Adaptation, from this perspective, involves more than emotional recovery. It also requires interpreting the event, redefining roles, and reconstructing expectations about family life after an unexpected disruption.

Despite growing recognition of the psychological and social consequences of trauma, relatively few studies have examined how relatives of traffic accident victims construct meaning while the injured person remains hospitalized. This gap is particularly relevant in Latin American settings, where available literature has largely emphasized epidemiological patterns, trauma systems, and clinical outcomes rather than the interpretive processes experienced by family members [10,11].

Grounded theory is appropriate for examining this phenomenon because it allows concepts and their relationships to emerge systematically from participants’ accounts rather than imposing a predetermined explanatory model [12]. The Straussian approach was adopted because its combination of constant comparison and axial coding offered a structured way to examine relationships among categories developed from the data, including causal conditions, contextual conditions, intervening conditions, actions/interactions, and consequences [13,14]. This analytical framework was particularly appropriate for exploring how relatives interpreted the accident while reorganizing caregiving roles and responding to uncertainty during hospitalization. Importantly, the Straussian coding paradigm was used to support conceptual integration after category development, not to impose predetermined categories or to assume that a single core category or fully developed substantive theory would necessarily emerge.

Accordingly, this study aimed to understand how family members of hospitalized traffic accident victims construct meaning around the accident during the patient’s hospitalization and to develop an empirically grounded conceptual account of this process.

## Materials and methods

Study design

This study used a qualitative grounded theory design. Grounded theory, originally developed by Glaser and Strauss [12], is an inductive methodological approach aimed at developing conceptual explanations of social processes through the systematic collection and analysis of empirical data. For methodological consistency, the study followed the Straussian analytical procedures developed by Strauss and Corbin [13,14], including open coding, constant comparison, analytical memo writing, and axial coding.

The Straussian approach was adopted because its combination of constant comparison and axial coding provided a structured means of examining relationships among categories developed from the data, particularly causal conditions, contextual conditions, intervening conditions, actions/interactions, and consequences. This analytical structure was appropriate for examining how family members interpreted the accident while reorganizing caregiving roles and responding to uncertainty during hospitalization. The Straussian coding paradigm was used to support conceptual integration after category development rather than to impose predetermined categories or to assume that a single core category or fully developed substantive theory would necessarily emerge.

Study setting

The study was conducted between February and October 2024 at a tertiary care hospital within Nicaragua’s social security system. In accordance with the institutional authorization granted for publication, the hospital is not identified by name in the manuscript to preserve institutional confidentiality.

Participants were recruited from inpatient wards caring for patients hospitalized following traffic accidents. Most hospitalized patients whose relatives participated in the study had traumatic injuries without an evident immediate threat to life, predominantly fractures of the upper or lower extremities. Although these injuries generally did not involve immediate life-threatening compromise, they produced relevant limitations in mobility, independence, and daily functioning and required inpatient care. No standardized injury severity classification was applied for this study.

Standardized information on injury severity, prognosis, hospitalization duration, neurological involvement, amputation risk, and perceived threat to life was not systematically collected under the original study protocol. Consequently, the analysis could not determine whether or how variation in these clinical characteristics influenced relatives’ meaning-making. This limitation should be considered when interpreting the findings and assessing their transferability, particularly to families of patients with substantially different trauma profiles or clinical trajectories, including severe neurological injury, prolonged hospitalization, major amputation risk, or an immediate threat to life.

Family involvement constituted an important component of the hospitalization experience examined in the study. Eligible relatives had assumed a caregiving role from the beginning of the event and remained actively involved in supporting the patient during hospitalization. This setting allowed meaning-making to be examined while family members were simultaneously confronting the emotional, practical, and caregiving consequences of the accident.

Participants and recruitment

Participants were recruited through criterion-based purposive recruitment according to predefined eligibility criteria. Eligible participants were adults, were immediate family members of a patient hospitalized following a traffic accident, had actively accompanied the hospitalization process from the initial event onward, and had assumed or directly participated in caregiving responsibilities.

After access to the inpatient wards was facilitated by hospital staff, the authorized interviewer independently identified potentially eligible relatives present in the wards and verified their eligibility according to the predefined criteria. Clinical and nursing staff did not identify, select, screen, or refer participants for inclusion; their role was limited to facilitating the researcher’s access to the inpatient areas. The interviewer approached eligible relatives directly and explained the purpose of the study, the voluntary nature of participation, confidentiality protections, and the interview procedures before obtaining informed consent. No formal screening log was maintained to document all potentially eligible individuals who were approached or who declined participation; consequently, the number of eligible individuals who declined participation cannot be reported reliably. Only individuals who met the eligibility criteria and provided informed consent were enrolled.

Recruitment and analysis proceeded concurrently. Formal theoretical sampling was not employed because the original study protocol established the participant group and eligibility framework in advance, and recruitment remained restricted to family caregivers meeting those predefined criteria rather than being redirected toward new participant profiles in response to emerging analytical categories. Saturation was therefore not used as an initial participant selection criterion. Instead, the need for additional interviews was assessed through concurrent analysis and constant comparison. As data collection progressed, each subsequent interview was examined for its contribution of new codes, properties, dimensions, variations, or relationships among the developing categories. Data collection ended after 10 interviews when later accounts continued to reinforce the emerging analytical structure without substantially modifying category properties or relationships. Saturation was thus used as the criterion for completing data collection rather than for selecting individual participants.

Additional variables such as educational level and socioeconomic status were not systematically collected under the original study protocol and were therefore not reconstructed retrospectively. Participant characteristics consistently available for analysis included age, gender, family relationship, and type of vehicle involved in the accident. Limitations related to the availability of clinical characteristics and standardized injury severity information are described in the Study setting section.

Data collection

Face-to-face, in-depth interviews were conducted using a semi-structured interview guide. The documented interview domains addressed family composition and living context; the circumstances in which participants learned about the accident and what occurred afterward; participants’ understanding and interpretation of the traffic accident; emotional responses since the event; support received from relatives, neighbors, and friends; consequences of the hospitalization for family life; experiences with healthcare personnel; perceptions of changes experienced by the injured family member; and expectations concerning what might occur after hospital discharge. These domains included both experiential and interpretive components and allowed participants to describe not only emotional distress and caregiving demands but also how they understood the event, attributed meaning to it, and anticipated its consequences for family life. The semi-structured format allowed the interviewer to use follow-up prompts when clarification or further elaboration was needed. All interviews were conducted by a single doctoral-level researcher who was formally authorized by the hospital to perform fieldwork within the institution. The interviewer was professionally affiliated with the hospital’s quality management structure and had responsibilities related to oversight of healthcare quality processes but had no direct clinical responsibility for the patients included in the study and did not participate in their medical treatment. Institutional authorization and security requirements restricted direct interviewing to this researcher. Interviews were conducted in a private space provided by the hospital, free from interruptions, and were audio-recorded after informed consent was obtained. Interview duration ranged from 24 minutes 30 seconds to 46 minutes 48 seconds, with a mean duration of approximately 36 minutes 5 seconds and a median duration of 36 minutes 27 seconds. Although the semi-structured format permitted participants to elaborate on issues they considered meaningful, the relatively short duration of some interviews may have limited the depth with which more complex or evolving dimensions of meaning-making and category relationships could be explored. This constraint was considered when defining the analytical product as an emerging conceptual model rather than a fully developed substantive theory.

Transcription and translation

All interviews were conducted in Spanish. Audio recordings were initially transcribed in Spanish using the automatic transcription function of Microsoft Word. The initial transcripts were subsequently verified against the corresponding audio recordings by two members of the research team. Transcription errors were corrected while preserving participants’ original wording, expressions, and intended meaning.

Only quotations selected for inclusion in the English-language manuscript were translated from Spanish into English. Bilingual members of the research team performed the translation, and the translated quotations were subsequently cross-checked against the original Spanish transcripts to ensure semantic and contextual equivalence. When literal translation could alter the intended meaning of a colloquial or context-specific expression, the wording was adapted to preserve conceptual meaning rather than strict word-for-word equivalence.

Researcher positionality and reflexivity

The research team included investigators with different institutional and academic backgrounds. The interviewer was the only member of the team professionally affiliated with the study hospital. The interviewer’s responsibilities involved oversight of healthcare quality processes but did not include direct clinical care of the patients or their relatives. The remaining researchers had no employment, clinical, or supervisory relationship with the study institution or participants and contributed primarily to the analytical and interpretive phases of the research.

The team recognized that the interviewer’s familiarity with the institutional environment and professional role in quality oversight could influence both the interview interaction and subsequent interpretation. Reflexivity was therefore incorporated throughout the analytical process. Emerging interpretations were discussed within the research team and repeatedly contrasted with the original interview data to distinguish participants’ accounts from researchers’ prior assumptions. The involvement of researchers without an institutional relationship with the hospital introduced additional perspectives during interpretive review and helped challenge assumptions potentially arising from familiarity with the study setting.

Data analysis

Verified Spanish-language transcripts were imported into ATLAS.ti 9 (ATLAS.ti Scientific Software Development GmbH, Berlin, Germany). Analysis followed Straussian grounded theory procedures involving open coding, constant comparison, progressive category development, analytical memo writing, and axial coding [13,14].

During open coding, interview transcripts were examined segment by segment, and initial conceptual codes were generated from participants’ accounts. Codes were continuously compared within individual interviews and across interviews to identify similarities, differences, recurring patterns, variations, and conceptual relationships. Through this constant comparative process, preliminary codes were refined, conceptually similar codes were grouped, and distinct phenomena were differentiated. These codes were progressively organized into broader concepts, categories, and subcategories.

Variation across participants’ accounts was retained as part of the analysis rather than assuming uniformity of experience. Accounts that differed from predominant patterns were compared with the developing categories and used to examine their conceptual boundaries and variability. A separate formal negative-case sampling strategy was not undertaken; instead, convergent, divergent, and discrepant accounts were considered within the constant comparative process to refine the developing categories and avoid forcing heterogeneous experiences into a uniform interpretation.

Analytical memos were written throughout the process to document emerging interpretations, conceptual relationships, questions, and analytical decisions. Axial coding was subsequently used to examine relationships among causal conditions, contextual conditions, intervening conditions, actions/interactions, and consequences. This stage enabled the researchers to move from predominantly descriptive codes toward an integrated conceptual explanation of how family members interpreted and responded to the accident during hospitalization.

The iterative analysis generated five interrelated categories: initial coping with the event; family reorganization and immediate overload; attribution of meaning to the accident; bodily vulnerability and empathy with the victim; and social support and the hospital environment. Because the analysis did not formally identify a single core category capable of integrating all categories into a fully developed substantive theory, the analytical product is presented as an emerging conceptual model of meaning-making rather than as a finalized substantive grounded theory.

Methodological rigor

Methodological rigor was addressed according to the criteria of credibility, transferability, dependability, and confirmability proposed by Lincoln and Guba [15].

Credibility was strengthened through verification of the transcripts against the original audio recordings, involvement of more than one researcher in transcript review and analytical interpretation, and repeated comparison of emerging interpretations with the original interview data. Constant comparison also allowed recurrent patterns to be examined alongside variation and discrepant accounts.

Transferability was supported through contextual description of the study setting, eligibility criteria, hospitalization context, caregiving involvement, and available participant characteristics, allowing readers to assess the relevance of the findings to other clinical settings and populations. At the same time, transferability should be interpreted in light of the absence of standardized clinical severity information and the specific trauma profiles represented in this study.

Dependability was strengthened through a systematic analytical sequence that progressed from transcription and open coding to constant comparison, category refinement, memo writing, and axial integration. Analytical decisions and emerging conceptual relationships were documented through memos, providing a traceable record of category development.

Confirmability was supported by maintaining a clear connection between interpretations and the original interview data, discussing emerging interpretations among researchers with different institutional relationships to the study setting, and examining divergent accounts during constant comparison. These procedures helped reduce the influence of prior assumptions and supported interpretations grounded in participants’ narratives.

Ethical considerations and data protection

The study was conducted after authorization was obtained from the hospital administration and the relevant teaching authorities. All participants received information about the purpose and procedures of the study and provided informed consent before participation. Particular attention was given to participants’ emotional well-being because the interviews addressed potentially distressing experiences related to traumatic injury and hospitalization.

The study was approved by the hospital’s institutional ethics committee under Minutes No. 001 of 2024, Code SCEHM-2024-01, dated January 5, 2024. The institution authorized publication of the study findings without disclosure of its identity; consequently, the hospital name and other directly identifying institutional details were omitted from the manuscript.

Access to study data was role-based. The interviewer was the only member of the research team with access to participants’ direct identities and to the linkage between personal identifiers and study codes. The interviewer and one additional member of the research team had access to the original audio recordings and complete transcripts for transcription verification and analytical purposes. The remaining researchers worked exclusively with coded and de-identified materials.

Audio recordings and transcripts were encrypted and password-protected. Identifying information was stored separately from the analytical dataset, and coded identifiers were used throughout the analytical process and manuscript preparation to minimize the possibility of identifying individual participants or linking them to the study institution.

## Results

A total of 10 family members of patients hospitalized following traffic accidents participated in the study. Participants ranged in age from 21 to 51 years and included eight women and two men. Family relationships comprised three wives, three mothers, two daughters, one son, and one brother. In nine of the 10 cases, the traffic accident involved a motorcycle; one case involved a bus (Table 1).

**Table 1:** General characteristics of the study participants. Source: Interviews with family members of traffic accident victims.

Family relationship	Age	Gender	Type of vehicle
Brother	23	Male	Motorcycle
Daughter	28	Female	Bus
Mother	42	Female	Motorcycle
Son	21	Male	Motorcycle
Wife	31	Female	Motorcycle
Wife	39	Female	Motorcycle
Mother	51	Female	Motorcycle
Daughter	35	Female	Motorcycle
Mother	46	Female	Motorcycle
Wife	36	Female	Motorcycle

Emerging conceptual model of meaning-making

Analysis generated five interrelated categories through which family members interpreted and responded to the traffic accident during hospitalization: initial coping with the event; family reorganization and immediate overload; attribution of meaning to the accident; bodily vulnerability and empathy with the victim; and social support and the hospital environment. These categories were not interpreted as isolated or strictly sequential experiences. Rather, they formed an interconnected process through which relatives progressively incorporated the accident into their understanding of caregiving responsibilities, family relationships, vulnerability, and expectations regarding the patient’s recovery and future.

Within this emerging conceptual model, notification of the accident and its perceived severity acted as the initial conditions that triggered emotional and cognitive disruption. Hospitalization, interpretations of responsibility, and the injured person’s bodily condition shaped the immediate context in which the event was understood. Family, community, religious, and healthcare support operated as intervening conditions, while family reorganization and caregiving decisions represented the principal actions and interactions undertaken in response to the event. Figure 1 summarizes these relationships.

**Figure 1:**
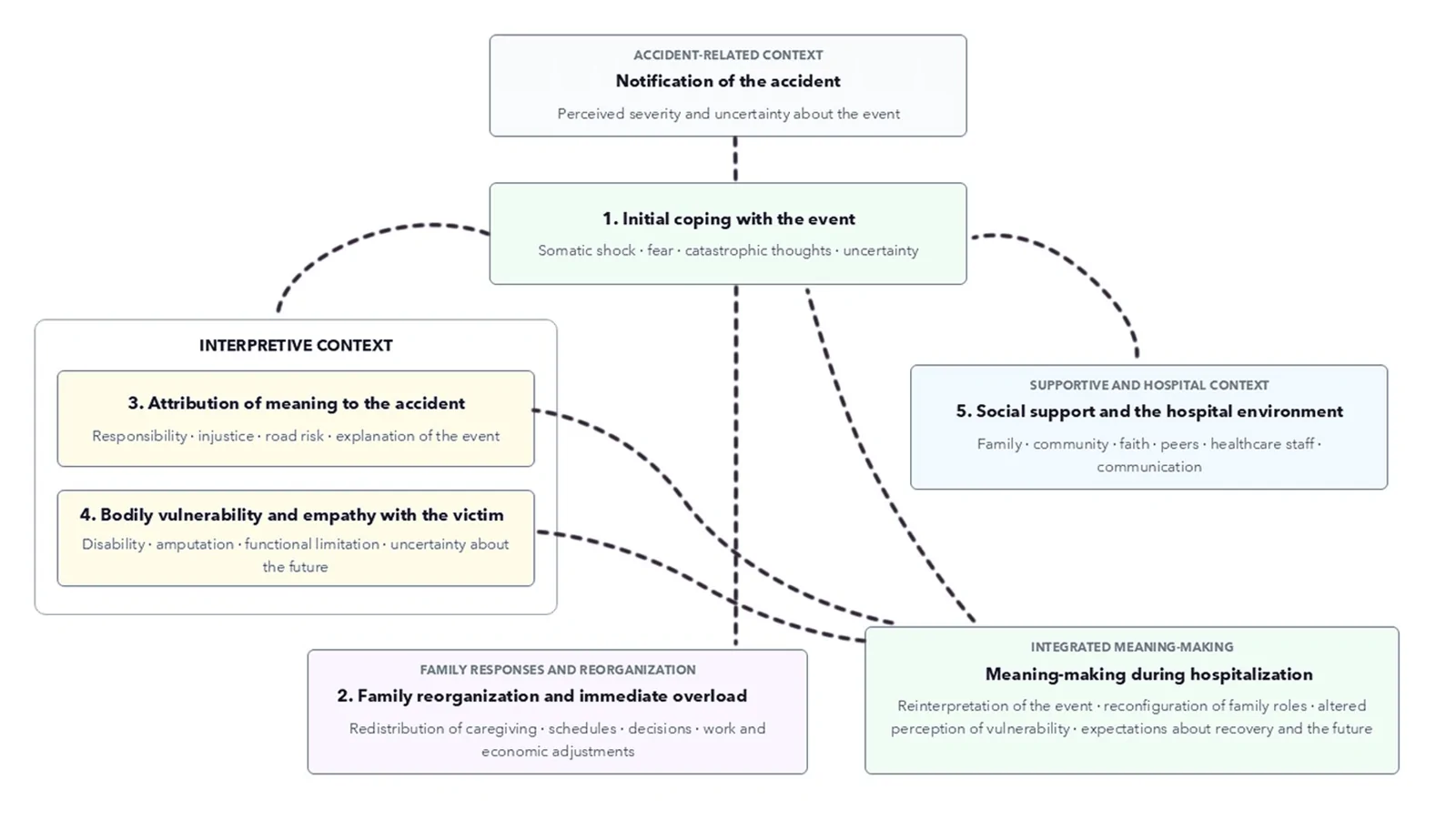
Emerging conceptual model of meaning-making during hospitalization among family members of traffic accident victims. The model illustrates conceptual relationships among the five analytical categories identified during hospitalization. Accident notification and perceived severity and uncertainty provide the context in which initial coping occurred. Attribution of meaning and perceived bodily vulnerability contributed to the interpretive context, while family reorganization reflected practical responses to hospitalization. Social, family, religious, and healthcare support formed part of the context in which these experiences were interpreted. The categories were interconnected rather than sequential or causal. Meaning-making during hospitalization represents an integrative interpretation of these relationships and does not constitute an additional category, a formally identified core category, or a demonstrated clinical outcome.

Initial coping with the event

Receiving notification of the accident marked an abrupt rupture in participants’ experience. Family members described immediate physiological and emotional reactions, including increased blood pressure, faintness, intense fear, loss of control, and catastrophic thoughts. Uncertainty intensified these responses. Before relatives fully understood the patient’s condition, they often interpreted the accident through the possibility of severe injury, disability, or death.

F3 stated, “Oh, no, I just wanted the ground to swallow me up … my blood pressure shot up and everything. Plus, I was thinking the worst.”

F8 recalled, “People I know who saw the accident called me, and I almost went crazy…”

F10 described the intensity of the immediate physical response: “When I look at that pool of blood, I almost pass out…”

The emotional shock did not remain separate from action. Relatives had to reach the hospital, seek information, coordinate with other family members, and begin organizing care while still experiencing fear and uncertainty. The accident therefore altered their position almost immediately: they moved from receiving unexpected news to assuming practical responsibilities linked to caregiving. This transition connected the initial emotional response with the family reorganization that followed.

Family reorganization and immediate overload

Hospitalization disrupted established routines and required rapid redistribution of family responsibilities. Participants described coordinating hospital attendance, dividing caregiving tasks, adjusting work schedules, managing transportation, and responding to new financial demands. These changes reorganized daily life around the injured person and created a form of immediate overload in which practical obligations accumulated while emotional distress remained present.

F1 explained, “After the call, I tried to talk to my sister to see what we were going to do… how we could support each other in caring for him.”

F6 described the economic consequences: “I lost my job… without a job, there’s less income, and living here is quite expensive.”

F7 stated, “I had to use all my vacation days so I could be here taking care of him.”

Family reorganization also changed how participants understood their own role within the household. Caregiving became associated with availability, presence, and the suspension or modification of other responsibilities. The accident thus acquired meaning not only through what had happened to the patient, but through what relatives now had to do in response. Because eight of the 10 participants were women, these caregiving experiences were predominantly narrated by women in this sample. This pattern reflects the composition of the study group and should not be interpreted as an estimate of caregiving distribution by gender in the wider population.

Attribution of meaning to the accident

Participants sought explanations for why the accident had occurred and what it represented. Their accounts incorporated responsibility, inattention, tragedy, vulnerability, and perceived risk on public roads. These interpretations gave the event a causal and moral structure. Rather than remaining an unexplained disruption, the accident became something participants attempted to locate within broader ideas about human error, danger, and the fragility of everyday life.

F1 stated, “For me, the accident is a lapse in attention… the driver’s fault.”

F2 described the event as, “It’s a tragedy… for the person who suffers it and for the person who causes it.”

F8 reflected on its disruptive potential: “It’s terrible how, in a minute, your life can be ruined… you’re just trying to live your life.”

These accounts reveal different ways of constructing meaning around the same type of event. Some relatives emphasized personal responsibility; others focused on tragedy or the sudden loss of control over ordinary life. Attribution therefore served as an interpretive mechanism through which participants organized the event within existing beliefs about responsibility, risk, and vulnerability.

Bodily vulnerability and empathy with the victim

This category captures how relatives interpreted the injured body and anticipated the consequences of physical damage. Unlike the somatic reactions described in the initial coping category, the emphasis here lies on the victim’s bodily condition and on what visible injuries represented for the future. Fractures, possible amputation, and uncertainty about functional recovery became concrete signs of vulnerability.

F3 stated, “When I saw him with his broken leg… I wondered if they were going to amputate his leg.”

F1 expressed uncertainty about future functioning: “Sometimes the injuries are to the head… will he be able to talk, walk, see you?”

F8 connected the present event with an earlier family experience: “I thought they were going to amputate his leg, just like they did with my other son.”

Participants did not interpret physical injuries only as clinical findings. They linked them to walking, communication, independence, and the possibility of permanent functional change. Visible bodily damage therefore shaped expectations about what the patient’s life, and, by extension, family life, might require after hospitalization. Previous traumatic experiences also influenced how some relatives interpreted the current event, intensifying anticipation of loss or disability.

Social support and the hospital environment

Participants relied on several forms of support during hospitalization. Family members shared caregiving responsibilities and expenses, while community and religious networks offered emotional reassurance. Peer interactions within the hospital also reduced feelings of isolation. These resources did not eliminate distress, but they changed how relatives managed it by distributing practical and emotional demands across a broader support network.

F3 stated, “People have been checking in on me… thank God, I haven’t felt alone.”

F10 emphasized collective involvement: “He hasn’t been left alone… we’ve been together through every decision.”

F8 described her experience with healthcare staff: “They’ve kept us informed about everything, and the nurses are kind.”

The hospital environment formed part of this support structure. Participants valued communication, respectful interactions, and explanations from healthcare professionals, particularly when uncertainty about treatment or recovery remained high. At the same time, prolonged hospitalization generated fatigue and a desire to return home. For some relatives, discharge represented more than leaving the hospital; it signaled a possible return to a more familiar environment in which the family could regain greater control over everyday life.

Integrative interpretation of the findings

Across the five categories, meaning-making emerged as a dynamic and interconnected process. The accident disrupted relatives’ emotional and cognitive equilibrium while family members simultaneously reorganized daily responsibilities, searched for explanations, interpreted the significance of the patient’s bodily injuries, and relied on social and institutional resources during hospitalization.

The findings distinguish the consequences of the accident from the process through which relatives interpreted those consequences. Emotional distress, work disruption, caregiving demands, bodily injury, and social support constituted important experiences in their own right. They became part of meaning-making when participants connected them with responsibility, family obligation, vulnerability, relationships, and expectations about recovery and the future.

Meaning-making therefore did not occur at a single moment. It developed as relatives progressively incorporated the accident into an altered understanding of the injured person, their own family role, and the immediate future. The five categories remained interconnected throughout this process rather than operating as strictly sequential stages.

## Discussion

The emerging conceptual model indicates that meaning-making during hospitalization developed through the interaction of interpretation, caregiving action, perceived vulnerability, responsibility, social support, and uncertainty. Previous qualitative research has shown that traumatic injury can disrupt family narratives, relationships, and expectations [16,17]. The present findings confirm this broader disruption but extend it by showing how relatives constructed meaning while the consequences of the accident were still unfolding. Rather than occurring as a later cognitive response, meaning-making developed alongside practical decisions, changing family roles, and attempts to understand an uncertain clinical future.

A central contribution of the model is therefore the distinction between experiencing consequences and assigning meaning to them. Emotional distress, occupational disruption, caregiving demands, bodily injury, and reliance on social support are already well documented in trauma and caregiving research. In the present study, these experiences became part of meaning-making when relatives connected them with responsibility, anticipated recovery, changing family roles, and possible future consequences. The model consequently emphasizes relationships among these dimensions rather than treating them as isolated consequences of hospitalization.

Initial coping with the event

Acute distress and uncertainty following traumatic injury have been previously described among injured individuals and their families [18,19]. The present findings confirm this immediate disruption but suggest that its conceptual importance lies in its coexistence with action. Relatives began seeking information, coordinating family responses, and assuming caregiving responsibilities before uncertainty about the injured person had been resolved.

Previous trauma-caregiving studies have similarly identified the tension between emotional distress and the practical requirement to act [20-22]. The present model adds that this transition also altered how relatives understood their own role. Becoming a caregiver, organizer, and information seeker was not merely a practical consequence of hospitalization; it became part of the process through which the accident acquired meaning. Interpretation and action therefore emerged concurrently rather than as separate sequential responses.

Family reorganization and immediate overload

Disruption of work, household responsibilities, social activities, and economic stability has been consistently reported among families caring for people with traumatic injuries [20,23]. The present study confirms these practical consequences. Its additional contribution lies in showing how family reorganization became an interpretive indicator of the magnitude of the event: relatives understood the accident partly through the extent to which ordinary family routines and responsibilities had to be reconfigured.

This relationship between disruption and interpretation strengthens the model beyond a description of caregiver burden. Changes in work and caregiving arrangements did not carry identical consequences for every participant, but collectively they demonstrated how hospitalization reorganized everyday family life. Meaning-making was therefore linked not only to what had happened to the injured person but also to what the accident required other family members to change.

Women predominated in the sample and frequently narrated caregiving responsibilities, consistent with previous qualitative evidence of substantial female involvement in post-injury care [23]. However, this finding should be interpreted as a characteristic of the present sample rather than evidence of a broader population-level gender distribution. The study contributes information about how these caregiving responsibilities were experienced, not about their comparative prevalence between women and men.

Attribution of meaning to the accident

Families confronting traumatic loss have also been shown to experience profound disruption and to incorporate the event into changing understandings of family life and future expectations [24,25]. The present findings confirm that relatives used causal and moral interpretations to make an abrupt event more understandable. However, these interpretations occurred while the outcome of the injury remained uncertain.

This unresolved temporal position differentiates the present findings from studies centered on traumatic death or completed recovery trajectories. Participants were constructing meaning while questions regarding functional recovery, disability, and future caregiving remained open. Previous trauma-caregiving literature has documented similar uncertainty regarding future clinical and functional trajectories [22]. The conceptual contribution here is that attribution was provisional: explanations of the accident developed alongside changing expectations about what the event might ultimately mean for the injured person and the family.

Bodily vulnerability and empathy with the victim

Previous qualitative studies have shown that changes in physical and functional capacity can alter relatives’ expectations, relationships, and perceptions of future family life [26]. The present findings confirm the importance of bodily injury but position physical vulnerability as a bridge between the immediate clinical event and anticipated changes in autonomy, mobility, and caregiving.

This relationship adds an important dimension to the model. The injured body was not interpreted solely in biomedical terms; it became a visible reference through which relatives imagined future dependence, recovery, or disruption of established family roles. Previous traumatic experiences could also influence these anticipations, suggesting that interpretations of present injury may be shaped by prior family experiences of bodily harm.

These findings remain limited to the hospitalization period. They support an interpretation of acute bodily and functional uncertainty but cannot establish whether these concerns persisted, diminished, or changed after discharge.

Social support and the hospital environment

Family and social support are well-established resources for individuals caring for relatives after traumatic injury [21]. The present findings confirm their practical and emotional importance but suggest an additional interpretive function. When caregiving responsibilities were shared, responsibility was experienced as distributed across a family or social network rather than located entirely in one individual.

Healthcare communication operated in a similar way. Previous studies have identified respectful communication, information, and professional support as important components of family experiences during trauma care [18,21]. In the present model, clinical information did more than provide reassurance. It helped relatives interpret bodily changes, treatment decisions, and uncertain expectations about recovery. Social support and healthcare communication therefore functioned not only as resources but also as intervening conditions shaping how vulnerability, responsibility, and uncertainty were understood.

The hospital environment also represented a temporary disruption of familiar routines. Expectations concerning discharge and return home reflected more than a change in location; they were associated with recovering familiarity, everyday control, and a sense of continuity. These interpretations should nevertheless be understood within the hospitalization period and not as evidence of adaptation after discharge.

Integration of the conceptual model

Taken together, the five categories support an interconnected rather than sequential account of meaning-making. Existing literature supports many of the individual components identified in the study, including emotional disruption, caregiving burden, family reorganization, attribution, bodily uncertainty, and social support [16-26]. The principal contribution of the present model lies in integrating these components within the acute hospitalization period and showing how they influenced one another while the outcome of the injury remained unresolved.

The model proposes that meaning emerged through the reciprocal interaction of interpretation and action. Relatives did not first experience the accident, subsequently adapt to caregiving, and only later construct meaning. Instead, meaning developed while they reorganized family life, assumed responsibilities, sought explanations, interpreted bodily vulnerability, and mobilized support. In this sense, caregiving actions themselves became part of the interpretive process.

This integration represents the main conceptual contribution of the study beyond previous descriptive literature. At the same time, the findings do not support claims of a fully developed substantive grounded theory. No single core category was formally identified, formal theoretical sampling was not undertaken, and the trauma profiles represented in the study were relatively circumscribed. The analytical product is therefore appropriately presented as an emerging conceptual model that organizes relationships observed during hospitalization and provides a basis for further theoretical development in more diverse trauma populations and across subsequent stages of recovery.

The small, context-specific sample and recruitment within a single hospital limit the transferability of the emerging conceptual model. In addition, interviews were conducted by a single researcher affiliated with the institution, which may have influenced participant selection, disclosure, or socially desirable responses despite the absence of a clinical relationship with participants and the reflexive procedures used during analysis. These factors should therefore be considered when assessing the strength and transferability of the conceptual relationships proposed by the model.

## Conclusions

This study identified an emerging conceptual model describing how family members of hospitalized traffic accident victims constructed meaning during the acute hospitalization period. Meaning-making developed through five interconnected categories: initial coping with the event; family reorganization and immediate overload; attribution of meaning to the accident; bodily vulnerability and empathy with the victim; and social support and the hospital environment. Rather than representing a strictly sequential process, these dimensions interacted as relatives confronted uncertainty, reorganized caregiving responsibilities, interpreted the causes and consequences of the accident, and reconsidered expectations about recovery and the immediate future of family life. Within this group, the accident extended beyond the injured person and altered relatives’ emotional responses, responsibilities, relationships, and perceptions of the immediate future. Caregiving experiences were predominantly narrated by women because eight of the 10 participants were female; this pattern reflects the composition of the sample and should not be interpreted as evidence of broader gender differences in caregiving. The findings therefore provide a context-specific understanding of family meaning-making rather than a universally generalizable account. From a clinical perspective, the findings generate hypotheses for family-centered trauma care by suggesting that relatives may benefit from clear communication, appropriate opportunities for involvement, and access to emotional and practical support during hospitalization. These implications should be interpreted as directions for future clinical research rather than evidence that such strategies improve patient or family outcomes.
